# Influence of Fermentation and Curing on Residual Nitrite, Physico‐Chemical, Volatile, Sensory, and Microbiological Properties of Sucuk Döner Kebab During Refrigerated and Frozen Storage

**DOI:** 10.1002/fsn3.72144

**Published:** 2026-07-29

**Authors:** Ayşe Sinan, Birol Kılıç, Azim Şimşek, Melis Yıldız

**Affiliations:** ^1^ Faculty of Engineering and Natural Sciences, Department of Food Engineering Suleyman Demirel University Isparta Türkiye; ^2^ Department of Food Processing Isparta University of Applied Sciences, Egirdir Vocational School Isparta Türkiye

**Keywords:** physico‐chemical, ripening, sensory, sucuk döner kebab, volatile compounds

## Abstract

The present study aimed to evaluate the influence of fermentation (starter culture addition), fermentation duration (24 and 72 h), and curing on volatile profile, physico‐chemical, sensory and microbiological features of sucuk döner kebab. Five treatments were prepared: one control (no starter cultures and curing) and four with starter cultures including two semi‐fermented (24 h) and two fermented (72 h) with and without curing. Fermentation reduced pH, with the lowest values observed in 72 h fermented treatments (*p* < 0.05). The highest cooking loss was obtained in uncured fermented treatments, whereas the lowest was found in their cured semi‐fermented counterparts (*p* < 0.05). Fermented treatments, particularly uncured ones, exhibited the lowest water activity (*p* < 0.05). L* values were similar among treatments, whereas cured treatments had higher a* and lower b* values than others (*p* < 0.05). Fermentation had a significant effect on reducing thiobarbituric acid reactive substances compared to control (*p* < 0.05). The highest residual nitrite was found in cured treatments (*p* < 0.05). Several volatile compounds, including 2‐methyl‐1‐butanol, allyl mercaptan and acetic acid, were dominant across treatments (*p* < 0.05). Fermented treatments, particularly those without nitrite, had higher acetaldehyde and ethanol. Sensory evaluations revealed that uncured semi‐fermented treatments were most preferred for overall acceptability (*p* < 0.05). Regardless of fermentation duration or curing, fermentation increased total mesophilic aerobic bacteria, lactic acid bacteria, and *Lactococcus‐Streptococcus* spp. counts compared to control (*p* < 0.05). Our findings demonstrated that fermentation, regardless of its duration or the curing process, played a crucial role in sucuk döner kebab production by improving its shelf life, quality, and safety.

## Graphical abstract

The effects of fermentation with and without nitrite on the physicochemical properties, lipid oxidation, volatile compounds, sensory characteristics, and overall quality of ready‐to‐eat sucuk döner kebabs were evaluated, demonstrating that fermentation, particularly without nitrite, improved oxidative stability and sensory quality.

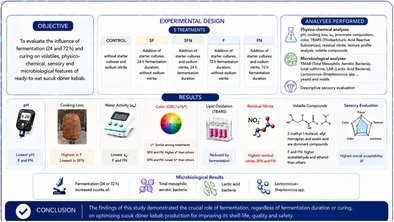

## 1 Introduction

A substantial increase in consumer demand for ready‐to‐eat (RTE) meat products has been observed. One of the most popular RTE meat products worldwide is döner kebab, which is very popular among consumers because it is easily accessible and tasty (Şimşek and Kılıç 2020). Döner kebab is a traditional Turkish meat product typically made from beef, lamb, or poultry meat, shaped into a cylindrical form around a skewer and subjected to rotational cooking either on a horizontal or vertical axis (Kilic 2009). In addition to döner kebab, sucuk, a traditional Turkish fermented sausage that has an important place in Turkish, Balkan, and European cuisines, is also frequently preferred by consumers (Yuca et al. 2019). Consumer expectations and the meat industry's efforts to create new meat products have resulted in the production of a new type of döner kebab called sucuk döner kebab, which includes some of the features of both sucuk and döner kebab. Sucuk döner kebab has strong potential for global appeal and widespread consumption. Sucuk döner kebab typically refers to a type of döner kebab where ground meat is seasoned or marinated with sucuk spices such as garlic, cumin, paprika, and red pepper flakes, attached to a doner skewer, cooked, sliced and served on plate or bread (Denktas et al. 2021). Sucuk döner kebab is often served in a similar fashion to traditional döner kebab, typically wrapped in flatbread or served with rice, salad, and various condiments such as yogurt sauce, pickles, and chili sauce (Gonulalan et al. 2004). Marinating or seasoning ground meat with spices typically found in sucuk creates a unique fusion of flavors, blending the savory taste of sucuk with the traditional döner kebab style of cooking. Döner kebab and sucuk döner kebab are also sold in packaged form, especially in grocery stores or supermarkets.

Even though sucuk is a fermented and cured meat product, the ground meat used in sucuk döner kebab manufacture is not subjected to fermentation or curing processes. Thus, sucuk döner kebab is a non‐fermented and uncured meat product. The absence of fermentation and curing steps may increase quality and safety concerns in RTE meat products because these processes contribute to microbial inhibition, oxidative stability, flavor development, and shelf‐life extension. Moreover, the lack of these preservation hurdles may increase susceptibility to spoilage and pathogen proliferation during storage, which is particularly important for packaged RTE products intended for prolonged storage, extended distribution chains, and commercial applications (Ye et al. 2025).

Fermentation is one of the oldest methods used in the long‐term preservation of muscle foods and provides the development of special taste and smell in the meat products (Leroy et al. 2013). Starter cultures, especially lactic acid bacteria (LAB), play an essential role in fermentation by inhibiting the proliferation of pathogenic and spoilage microorganisms, thereby improving the microbiological safety of the end product (Ertürkmen et al. 2016). In addition to improving safety and product quality, starter culture application may also provide technological and economic advantages by facilitating process standardization, reducing production losses, and enabling integration of fermentation with transportation and distribution stages, thereby potentially improving processing efficiency and lowering overall production costs. Furthermore, fermentation duration may markedly affect acidification kinetics, microbial ecology, volatile compound formation, and sensory characteristics, thereby influencing the final quality attributes of fermented meat products (Siddiqui et al. 2023; Kaya et al. 2025). On the other hand, nitrites and nitrates are widely used ingredients in the processing of muscle foods due to their antimicrobial and antioxidant effects as far as their influence on the formation of characteristic cured color and flavor in the meat products (Kilic et al. 2001). Nitrite and nitrate use in fermented meat products should be carefully optimized, as starter cultures can also contribute to color formation during fermentation. However, their effect depends on strain‐related characteristics such as nitrate‐reductase activity, growth characteristics, and acid tolerance; therefore, appropriate starter culture selection is important for targeted color development (Gøtterup et al. 2008). The curing process may also influence residual nitrite levels, oxidative stability, volatile profile, sensory quality, and microbial safety of meat products during storage. Therefore, evaluation of residual nitrite is particularly important because it reflects both product safety and curing efficiency while also being associated with consumer concerns regarding nitrite intake. Likewise, volatile compounds are closely related to flavor development and consumer acceptance, whereas physicochemical, microbiological, and sensory properties collectively determine product stability, shelf life, and marketability (Ye et al. 2025). Producing under hygienic conditions and investigating the chemical quality of döner kebab has become a subject on which the meat industry and researchers focus. The studies conducted focus on innovative approaches and researching different techniques to extend the shelf life of RTE döner kebabs and increase food safety and quality (Kilic 2009). Recent studies have increasingly focused on improving the quality, safety, and storage stability of RTE meat products through fermentation‐based strategies and optimization of curing conditions. However, information regarding the application of these approaches to sucuk döner kebab remains very limited.

There is limited information in the literature on sucuk döner kebab, which is a new type of döner kebab. For this reason, more studies need to be carried out on sucuk döner kebab to generate valuable knowledge for enhancing the safety and quality attributes of this highly demanded meat product. No previous study has comprehensively evaluated the combined effects of fermentation, fermentation duration, and curing on residual nitrite, volatile profile, physicochemical characteristics, sensory properties, and microbiological quality of RTE sucuk döner kebab during both refrigerated and frozen storage. Therefore, investigation of these parameters may provide important scientific insights and practical guidance for improving product safety, quality, and industrial applicability.

This study aimed to evaluate the influences of fermentation, fermentation duration, and curing process on residual nitrite, volatile profile, physico‐chemical, sensory, and microbiological properties of RTE sucuk döner kebab during refrigerated and frozen storage.

## 2 Materials and Methods

### 2.1 Materials

*M. longissimus thoracis et lumborum* muscles (24 h post mortem) from the carcasses of two cattle (Simmental, approximately 1.5 years old) and beef back fat were obtained from a local supplier (Isparta, Türkiye) for each replication. Meat and beef back fat purchase was repeated three times in the same manner on separate processing days for each batch of three independent replications. A total of six cattle carcasses were used throughout the entire study. The muscles were transported to the laboratory in refrigerated conditions and then all visible external fats and connective tissues were trimmed off. After grinding and vacuum packaging, the loin meat was stored frozen (−20°C) until used. The beef back fat was ground, vacuum packed, and stored at −20°C until needed for sucuk döner kebab preparation. Spices and the mixture of starter cultures (BAKTOGARDSW‐10) were obtained from Bağdat Spices (Ankara, Türkiye) and Inovative Biotechnology Chemistry and Health Ltd. (İstanbul, Türkiye), respectively.

### 2.2 Sucuk Döner Kebab Preparation and Design of Treatments

**FIGURE 1:**
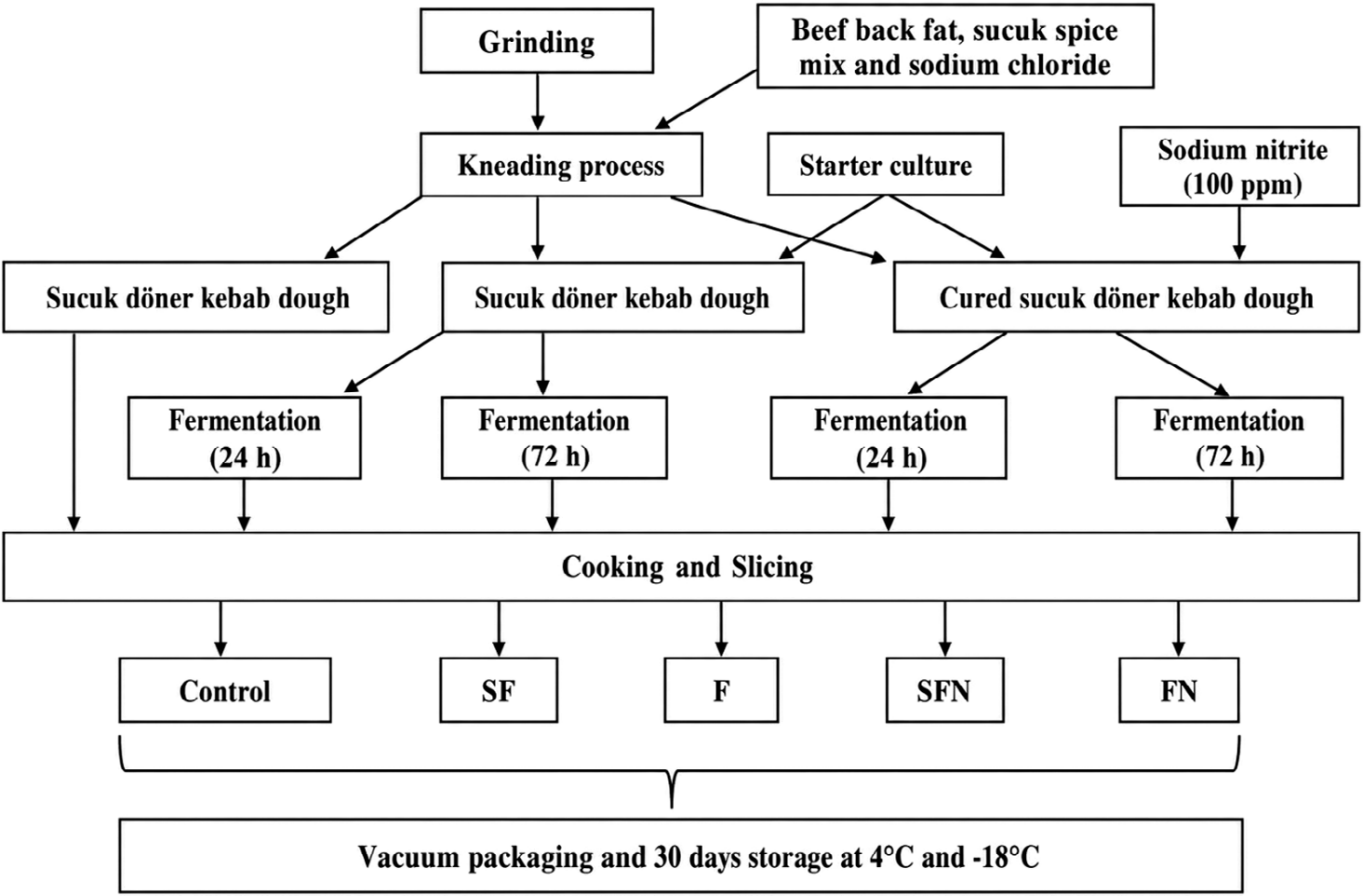
Process flow chart of sucuk döner kebabs. Control, Sucuk döner kebab (without starter cultures and sodium nitrite); F, Fermented sucuk döner kebab (addition of starter cultures and 72 h fermentation duration); FN, Fermented sucuk döner kebab with 100 ppm added sodium nitrite; SF, Semi‐fermented sucuk döner kebab (addition of starter cultures and 24 h fermentation duration); SFN, Semi‐fermented sucuk döner kebab with 100 ppm added sodium nitrite.

The formulation and preparation of sucuk döner kebab dough was performed as described by Özer and Kılıç (2020). After thawing and first grinding (9.5 mm) of the meat, 25% beef back fat was added and then the mixture was reground (3.2 mm) in a meat grinder. The basic sucuk döner kebab dough was prepared without incorporation of starter culture and sodium nitrite and contained ground lean beef (75%) and beef back fat (25%). A 2% sodium chloride (Merck, Germany), 6% distilled water, 0.6% saccharose, 1.5% garlic, 1.5% sweet red pepper, 0.5% hot red pepper, 0.5% black pepper, 0.75% cumin, and 0.25% allspice were also used in the production of all sucuk döner kebab dough. The proportions of these ingredients were calculated based on the total weight of ground lean beef and beef back fat. After the kneading process, sucuk döner kebab dough was divided randomly into 5 batches with equal weights (approximately 2 kg each). The present study involved the production of five different RTE sucuk döner kebabs, including one control sucuk döner kebab produced without addition of starter cultures and sodium nitrite, two semi‐fermented (addition of starter cultures and 24 h fermentation duration) sucuk döner kebabs with or without added sodium nitrite (100 ppm), and two fermented (addition of starter cultures and 72 h fermentation duration) sucuk döner kebabs with or without added sodium nitrite (100 ppm). Then, the mixture of starter cultures (*Lactiplantibacillus plantarum* and *Lactobacillus sakei* ; 10^8^ CFU/g) was incorporated into semi‐fermented and fermented sucuk döner kebab doughs. In addition, 100 ppm sodium nitrite (Merck, Germany) was also added to one semi‐fermented and one fermented sucuk döner kebab treatments which were designed to have sodium nitrite addition. The semi‐fermented and fermented sucuk döner kebab samples were processed in a digital fermentation chamber (TK‐252, Nüve, Ankara, Türkiye) equipped with a temperature and humidity control unit. Fermentation was carried out at 20 C and 95% relative humidity for 24 h for the semi‐fermented treatments and 72 h for the fermented treatments. Heat treatment, slicing, and vacuum packaging were applied to all sucuk döner kebabs as described by Şimşek and Kılıç (2020). Each sucuk döner kebab batch was positioned 10 cm in front of an open gas oven. Cooking was conducted under an exhaust hood, and the heating intensity was regulated via a gas valve to ensure consistent thermal exposure. Each surface of the cylindrical sucuk döner kebab batch was cooked for 4 min before being rotated. After each surface was cooked, the outer layer was shaved off in thin slices (approximately 5 mm thick) by an electric handheld döner slicer. This process was repeated until the entire sucuk döner kebab batch was cooked and sliced. The slices were then vacuum‐packaged in polyamide/polyethylene bags with an oxygen permeability of 10 cm^3^/(m^2^·24h·0.1 MPa) using a vacuum packaging machine (Ramon VP280, Barcelona, Spain). Then, RTE sucuk döner kebab samples were stored at either 4°C (refrigerated) or −18°C (frozen) for 30 days. Whole sucuk döner kebab production process is presented by a schematic diagram (Figure 1). Sucuk döner kebab slices were separated randomly for analysis. Each experiment was independently replicated three times, and within each replication, three samples from each sucuk döner kebab treatment were analyzed for all tested parameters. Various physico‐chemical, sensory, textural, and microbiological properties were assessed. Sampling was conducted on processing day (day 0) for cooking loss, water activity, proximate composition, color, texture, pH, thiobarbituric acid reactive substances (TBARS), residual nitrite, volatile compound profile, sensory evaluation, and microbiological analysis (total aerobic mesophilic bacteria, lactic acid bacteria, coliform group bacteria, *Lactococcus‐Streptococcus* spp., and total mold and yeast count). Moreover, water activity, color, pH, TBARS, and microbiological analysis were performed at 15 and 30 days of storage. In addition, residual nitrite analysis was carried out at the end of storage (day 30). For each time point and batch, three replicate samples were collected, and all analyses were carried out accordingly.

### 2.3 Cooking Loss Analysis

The weight of the raw sucuk döner kebab dough used for each treatment was recorded. Calculation of weight loss after cooking in sucuk döner samples was assessed after the samples were allowed to cool to around 25°C. Cooking loss was calculated according to the formula shown below:

(1) $$\text{Cooking loss}\;\left(\%\right)=\frac{\left(\mathrm{raw}\;\text{sucuk döner kebab dough weight}-\text{cooked sucuk döner kebab weight}\right)}{\mathrm{raw}\;\text{sucuk döner kebab dough weight}}\;x\;100$$

### 2.4 Determination of pH, Water Activity (a_w_), Color, Texture and Proximate Composition

pH values of sucuk döner kebabs were measured at ambient temperature by a pH meter (HI 2211, Hanna Instruments, Woonsocket, RI, USA) (Soyuçok et al. 2024). Calibration of the pH meter was carried out with pH 4.0 and 7.0 buffer solutions. The a_w_ values of sucuk döner kebabs were evaluated at 25°C by a water activity device (Novasina LabSwift‐a_w_, Lachen, Switzerland) in triplicate (Tenderis et al. 2021). Sucuk döner kebabs were subjected to color measurements by using Precise Color Reader TCR 200 (BAMR Ltd., Claremont, South Africa) which had an illuminant D65, 10° standard observer position and 8 mm aperture size. After holding the samples at room temperature for 10 min, color values (CIE L*, a*, and b*) were measured on the randomly choosen locations at both surfaces of sucuk döner kebab samples during storage period (Uşan et al. 2022). Texture profile analysis (TPA) of cooked sucuk döner kebab samples was performed in triplicate for each treatment at each replication by a CT3 Texture Analyzer (Brookfield Engineering Lab Inc. U.S.A.). The TPA parameters calculated from the force‐time curves included hardness, adhesiveness, cohesiveness, springiness, chewiness, and resilience. Texture measurements were performed with rectangular aluminum probe (9 mm × 35 mm × 0.05 mm) for compression and blade for cutting (25 kg load cell). A double compression cycle was applied to simulate mastication and determine textural parameters. Texture analysis test conditions were: pre‐test speed of 1.0 mm/s, test speed of 3.0 mm/s, post‐test speed of 10 mm/s, and compression level of 70% (Kılıç and Özer 2019). Proximate composition including the moisture, protein, fat and ash contents was determined with respect to the AOAC (2005) methods.

### 2.5 Thiobarbituric Acid Reactive Substances (TBARS) Analysis

TBARS measurements were carried out three times for each sucuk döner kebab sample, in accordance with the methodology described by Soyuçok et al. (2024). The procedure involved blending 1 g of sucuk döner kebab sample with trichloroacetic acid (6 mL) extraction solution containing EDTA and propyl gallate. The mixture was homogenized and filtered, and then 1 mL of the filtrate was combined with 1 mL of thiobarbituric acid. The resulting mixture was then heated at 100°C for 40 min. After centrifugation and cooling, the absorbance was measured at 532 nm against a reference solution of 1 mL trichloroacetic acid and 1 mL thiobarbituric acid. The results were expressed as μmol MDA per kilogram of sucuk döner kebab sample.

### 2.6 Residual Nitrite Analysis

A mixture consisting of sucuk döner kebab samples (approximately 5 g) and 40 mL distilled water (80°C) was prepared, followed by the addition of 350 mL distilled water and subsequently boiled in a water bath for 2 h with occasional stirring. Then, the samples were cooled to ambient temperature, and the volume was adjusted to 500 mL using distilled water. Mercuric chloride (5 mL) was incorporated into the sample solution and homogenized, followed by cooling to ambient temperature and filtration, after which 2 mL of Griess reagent was added to the filtrate and mixed thoroughly. The mixture was incubated in dark for 1 h to allow color development, followed by measurement of absorbance at 520 nm, and residual nitrite concentrations were calculated from the formula generated from the standard curve and adjusted using a dilution factor 20 (AOAC 2000). The limit of detection (LOD) and limit of quantification (LOQ) for nitrite were 0.4 mg/kg and 1.3 mg/kg, respectively. The results were reported as mg of residual nitrite per kg of sucuk döner kebab sample.

### 2.7 Microbiological Analysis

Sucuk döner kebab samples were analyzed for total mesophilic aerobic bacteria (TMAB), total coliform bacteria, total yeasts and molds using methods described by Köker et al. (2024). In order to determine TMAB, total coliforms, and total yeasts and molds, Plate Count Agar (PCA, Merck, Germany), Eosin Methylene‐blue Lactose Sucrose (EMB, Merck, Germany) Agar, and Potato Dextrose Agar (PDA, Merck, Germany) were used as selective agar mediums, respectively. TMAB and total coliform bacteria counts were enumerated on PCA after incubation at 30°C for 24–48 h and EMB agar medium after incubation at 37°C for 24–48 h, respectively. Total yeasts and molds were enumerated on PDA after incubation at 25°C for 72–120 h. LAB and *Lactococcus‐Streptococcus* spp. counts were determined using the double‐layer technique on de Man, Rogosa and Sharpe (MRS) agar (Merck, Darmstadt, Germany) and M17 agar (Merck, Darmstadt, Germany), respectively (Comi et al. 2005). MRS and M17 agar mediums were incubated at 30°C for 72 h. Results are presented as Log CFU/g of sucuk döner kebab sample.

### 2.8 Volatile Profile Analysis

The amounts of qualitative volatile compounds in sucuk döner kebabs were determined using a gas chromatography–mass spectrometry system (Shimadzu GC–MS‐QP2010 SE, Shimadzu Corporation, Kyoto, Japan) coupled with solid phase microextraction. Three grams of homogenized sucuk döner kebab samples were transferred to 15 mL headspace tubes (clear PTFE/Silicone septa Cap) with a solid phase microextraction injector (Fused silica SPME fiber CAR/PDMS) and the tubes were kept at 60°C for 45 min. Volatile compounds released during waiting were injected into the Restek Rx‐5Sil MS (30 m‐0.25 mm‐0.25 μ m) column from the SPME injector. After the column was kept at 40°C for 2 min, it was kept at 250°C for 5 min after the temperature increased to 250°C with an increase of 4°C per min. Helium gas, which was used as carrier gas in the analysis, was passed through the column at a flow rate of 1.61 mL/min. The injector temperature and inlet pressure were 250°C and 83.5 kPa, respectively. Other analyses conditions were: injection mode, split (10:1); MS interface temperature, 250°C; MS mode, electron ionization; detector voltage, 1.5 kV; mass range, 35–450 m/z; scan speed, 1428 u/s; interval, 0.30 s (2 Hz). GC/MS analysis was performed in scan mode over a mass range of 40–300 amu. The peaks obtained during the analysis were identified by scanning the Wiley 7 library, and the calculation of the retained volatile compounds was performed by dividing the peak areas obtained from the GC–MS analysis by 10^6^ (Corral et al. 2015).

### 2.9 Sensory Evaluation

The test panel for five sucuk döner kebab treatments (each represented by one slice and placed together on the same plate) was conducted for each repetition and repeated three times on separate processing days. In total, three separate test panel sessions were conducted on three different processing days. Sensory evaluation of sucuk döner kebab samples was performed in a specially prepared room for sensory analysis by 25 panelists from the Food Engineering Department at the Süleyman Demirel University. The panel included academic staff and graduate students who are experienced in the sensory assessment of muscle foods. All participants provided informed consent before their involvement in the sensory evaluation. Sucuk döner kebab samples for each treatment were served to the panelists on white plastic plates labeled with randomly assigned three‐digit codes. Sucuk döner kebab samples underwent descriptive sensory evaluation for color intensity, color uniformity, juiciness, integrity, texture, greasiness, off‐flavor, off‐odor, and overall acceptability using a 9 cm linear scale (Uysal et al. 2022). For all attributes, the scale ranged from 1 to 9, with the anchor points defined as follows: color intensity (extremely light to extremely dark), color uniformity (not uniform to uniform), juiciness (dry to juicy), integrity (fragmented to not fragmented), texture (extremely soft to extremely hard), greasiness (not greasy to extremely greasy), off‐flavor and off‐odor (not detected to extremely high), and overall acceptability (extremely low to extremely high).

### 2.10 Statistical Analysis

Entire study was repeated as three replications which were conducted on separate processing days and analysis carried out for each replication were also performed in triplicate. To determine the effect of fermentation, fermentation duration and curing process on the dependent variables (residual nitrite, volatile compounds, physico‐chemical, sensory and microbiological properties), the study employed a completely randomized design comprising five treatments which included control (without addition of starter cultures and sodium nitrite) and four treatments (Semi‐fermented sucuk döner kebab, SF; semi‐fermented sucuk döner kebab with 100 ppm added sodium nitrite, SFN; fermented sucuk döner kebab, F; fermented sucuk döner kebab with 100 ppm added sodium nitrite, FN). Generalized linear model (GLM) was assessed on the collected data to determine significant difference for each dependent variable during certain intervals of storage periods. The data for pH, a_w_, color, texture, TBARS, and microbiological analyses were evaluated using a 5 × 3 factorial design, while the data for residual nitrite were analyzed using a 5 × 2 factorial design; both models included two factors: treatment and storage time. In the statistical model, treatments, storage times and the interaction term between these two factors were designated as fixed effects, and replicates were included as a random effect. These statistical models were applied independently to the data collected from samples stored at refrigerated and frozen temperatures for all the analyses mentioned above. Thus, the effects of storage type (refrigerated versus frozen) difference on the evaluated parameters were not assessed in this study. Moreover, the data obtained from cooking loss, volatile compounds, proximate composition and sensory evaluation were subjected to one‐way analysis of variance (ANOVA). For cooking loss, volatile compounds and proximate composition, the treatments were included as a fixed effect, while the replicates was treated as a random effect. The statistical evaluation was carried out using Minitab 19.1.1. Statistical differences among means were assessed using Tukey's multiple comparison test, with significance defined at *p* < 0.05. The results were presented as mean values ± standard error (SE). Principal component analysis (PCA) was performed independently for treatments stored at 4°C and −18°C to evaluate the relationships among TBARS, pH, a_w_, residual nitrite, color, and microbiological parameters. PCA results were interpreted based on eigenvalues, explained variance, and cumulative variance derived from the correlation matrix of the analyzed parameters. Principal components with eigenvalues greater than 1 were retained for interpretation. Factor loadings were used to determine the contribution of each parameter to the principal components, and PC1–PC2 biplots were generated to visualize the relationships among treatments and parameters. Separate PCA analyses were performed for treatments stored at 4°C and −18°C using GraphPad Prism 11 (GraphPad Software, Boston, MA, USA).

## 3 Results and Discussion

### 3.1 Cooking Loss

Results (Table 1) revialed that the highest cooking loss occurred in F treatment while the lowest cooking loss occurred in SFN treatment (*p* < 0.05). SFN treatment was followed by SF treatment. Control and FN treatments had similar cooking loss values. A key factor driving these differences are thought to be pH values of each döner kebab treatments, which is closely linked to the water retention capacity of the meat, ultimately influencing cooking loss. In general, lower pH (more acidic environments) reduce the water‐holding capacity of muscle proteins, leading to higher cooking loss (Xiong et al. 1999). In the present study, F treatment exhibited the lowest pH and the highest cooking loss values (*p* < 0.05). Fermentation typically leads to a pH reduction due to the accumulation of organic acids produced by lactic acid bacteria (Punia Bangar et al. 2022). As pH drops, the isoelectric point of muscle proteins is reached, causing proteins to lose their ability to bind water effectively, resulting in increased cooking loss (Goemaere et al. 2021). Therefore, it is thought that the reason for the highest cooking loss in F treatment is related to the decrease in the water holding capacity of the meat in an acidic environment. A similar situation is also valid for FN treatment, which also had low pH. In this concept, the finding the lowest cooking loss in SFN treatment could be attributed to the more basic pH relative to the other fermented treatments. Semi‐fermented products typically have a moderate pH, not as low as fermented products, which helps retain more water in the muscle during cooking (Barbut 2006). In contrast, SF treatment showed a slightly higher cooking loss values than SFN treatment, but still lower than the fermented treatments (F and FN) (*p* < 0.05). On the other hand, control, which was not fermented, did not exhibit the same inverse relationship between pH and cooking loss, highlighting the complex role that fermentation plays in modifying the structure and water‐holding capacity of meat. This finding indicates that factors other than pH also influenced cooking loss. Fermentation‐induced proteolysis and modifications in the protein matrix may alter water‐holding properties independently of pH, whereas such structural changes were absent in control (Candogan et al. 2009; Wang, Cheng, et al. 2022; Wang, Yu, et al. 2022).

**TABLE 1:** Cooking loss and proximate compositions of sucuk döner kebab treatments.

Treatments	Cooking loss (%)	Moisture (%)	Fat (%)	Protein (%)	Ash (%)
Control	35.51^b^	44.67^ab^	19.56^c^	31.24^a^	4.02^ab^
SF	33.78^c^	41.46^b^	22.09^ab^	31.97^a^	4.06^ab^
F	37.75^a^	42.10^b^	20.66^abc^	32.54^a^	4.15^a^
SFN	32.15^d^	47.24^a^	19.86^bc^	29.13^a^	3.48^c^
FN	36.12^b^	43.57^ab^	22.92^a^	29.47^a^	3.74^bc^
SEM	0.44	1.15	0.58	0.85	0.08

*Note:* ^a–d^Within a column, values superscripted with different letters are significantly different (*p* < 0.05).

Abbreviations: Control, sucuk döner kebab (without starter cultures and sodium nitrite); F, fermented sucuk döner kebab (addition of starter cultures and 72 h fermentation duration); FN, fermented sucuk döner kebab with 100 ppm added sodium nitrite; SEM, standard error of the mean; SF, semi‐fermented sucuk döner kebab (addition of starter cultures and 24 h fermentation duration); SFN, semi‐fermented sucuk döner kebab with 100 ppm added sodium nitrite.

### 3.2 pH, a_w_, Color, Texture and Proximate Composition

Results (Table 2) indicated that the observed pH trends in this study were strongly influenced by the fermentation process (*p* < 0.05). Among the sucuk döner kebab samples prepared for both +4°C and −18°C storage, the control exhibited the highest pH values both on the processing day and after 30 days storage (*p* < 0.05). On the other hand, on processing day, the lowest pH levels among sucuk döner kebab samples prepared for both +4°C and −18°C storage were in the fermented sucuk döner kebab samples (F and FN, *p* < 0.05). These findings demonstrated that the fermentation process plays a significant role in reducing pH in fermented samples, which is typically attributed to the formation of organic acids, such as lactic acid, which are by‐products of the fermentation (Ashaolu et al. 2023). Although only acetic acid was detected in the volatile profile, this does not indicate the absence of other organic acids, particularly lactic acid. Due to its limited volatility, lactic acid and formic acid may not be reflected in the volatile profile (Olivares et al. 2015). Therefore, the pH reduction observed in F and FN treatments was still considered to be associated with LAB metabolism and the production of non‐volatile organic acids during fermentation. Moreover, it was determined that the semi‐fermented sucuk döner kebab samples (SF and SFN) exhibited lower pH values than the control and higher pH values than the fermented sucuk döner kebab samples (*p* < 0.05). This result suggested that the extent of fermentation influences the rate of pH reduction. This finding aligns with previous studies on fermented sausages, where intermediate fermentation processes lead to pH levels that are lower than non‐fermented sausages but higher than those of fermented products (Afifah et al. 2023). These observations imply that partial fermentation can offer a balance between desired flavor and texture changes without excessively lowering pH. Furthermore, our results showed that the nitrite addition had no effect on the pH values of sucuk döner kebab samples prepared to be stored at both +4°C and −18°C. This finding is consistent with some previous research indicating that nitrites primarily affect color and microbial inhibition rather than directly altering pH of cured meat products. While nitrites are essential in controlling pathogenic bacteria and enhancing the color of processed meats, they do not appear to participate in acidification during the initial stages of fermentation (Wang, Cheng, et al. 2022; Wang, Yu, et al. 2022). During storage at +4°C, pH values in SF and SFN treatments decreased gradually (*p* < 0.05). On the other hand, there was no pH change in the other treatments. Regarding the samples stored at −18°C, there was no pH change in the treatments except FN, where pH values increased gradually (*p* < 0.05). These findings revealed that the storage temperatures (+4°C and −18°C) also impacted pH. While refrigeration at +4°C is known to slow down fermentation, freezing at −18°C typically inhibits the activity of starter cultures and, consequently, pH changes. This suggests that the fermentation process is inhibited by freezing temperature, and pH remains higher, as observed in control. These findings contribute to the understanding of how fermentation and storage conditions interact to influence the quality of sucuk döner kebab. This result is consistent with studies showing that lower temperatures slow the growth of lactic acid bacteria, which are primarily responsible for pH reduction during fermentation (Sionek et al. 2024). In the present study, lactic acid was not quantified due to the analytical approach used for volatile analysis; therefore, the interpretation of pH reduction was based on microbial activity and fermentation extent rather than direct lactic acid determination (Olivares et al. 2015). In contrast, at −18°C, pH values in the control also remained high after 30 days, suggesting that freezing halts microbial activity almost completely, thus preventing further acidification. As freezing maintains the microbial stability of the product while suppressing fermentation processes, the control samples exhibited a nonsignificant reduction in pH values. When pH values after 30 days storage at +4°C were compared, the highest pH values were obtained in the control as determined on the processing day. The lowest pH levels were obtained in F and FN treatments (*p* < 0.05) and the values determined in SF and SFN were between control and fermented sucuk döner kebab treatments. In these samples, since fermentation was not as extensive, they had a moderate pH drop compared to the control but did not reach the low levels seen in fermented samples. While the nitrite addition did not cause a pH difference in F and FN treatments during 30 days storage at +4°C, it caused a difference in pH in SF and SFN treatments (*p* < 0.05). The results indicated that pH values of SF were lower than SFN (*p* < 0.05). In the samples stored at −18°C, the highest pH values were in the control after 30 days storage. The lowest pH values were detected in the F treatment, followed by the FN treatment (*p* < 0.05). Similar to +4°C, pH values detected in SF and SFN treatments were lower than control and higher than those in F and FN treatments (*p* < 0.05). Unlike storage at +4°C, higher pH values were detected in the nitrite added treatments in both fermented and semi‐fermented sucuk döner samples after 30 days storage at −18°C (*p* < 0.05).

**TABLE 2:** pH; water activity (a_w_) and TBARS results of the cooked sucuk döner kebab treatments stored at 4°C and −18°C.

	Treatments	Storage time (days; +4°C)			Storage time (days; −18°C)		
		0 days	15 days	30 days	0 days	15 days	30 days
pH	Control	5.61^a^	5.64^a^	5.57^a^	5.59^a^	5.64^a^	5.65^a^
	SF	5.36^b^	5.31^bc^	5.16^d^	5.35^c^	5.37^bc^	5.35^c^
	F	4.52^e^	4.56^e^	4.49^e^	4.53^f^	4.51^f^	4.55^ef^
	SFN	5.39^b^	5.38^b^	5.26^c^	5.37^bc^	5.39^bc^	5.42^b^
	FN	4.50^e^	4.51^e^	4.55^e^	4.52^f^	4.60^de^	4.61^d^
	SEM	0.03			0.02		
Water activity (a_w_)	Control	0.81^f^	0.84^a–d^	0.85^abc^	0.81^de^	0.83^abc^	0.84^ab^
	SF	0.85^a–d^	0.84^bcd^	0.86^a^	0.84^a^	0.83^a–d^	0.84^a^
	F	0.80^g^	0.81^f^	0.83^de^	0.80^e^	0.83^a–d^	0.83^a–d^
	SFN	0.84^bcd^	0.84^bcd^	0.85^ab^	0.84^abc^	0.84^ab^	0.84^ab^
	FN	0.82^ef^	0.83^de^	0.84^cde^	0.82^bcd^	0.82^cde^	0.82^cde^
	SEM	0.006			0.007		
TBARS (μmol MDA/kg)	Control	2.37^b^	2.59^b^	3.23^a^	2.51^ab^	2.34^b^	2.77^a^
	SF	1.48^cd^	1.56^c^	1.49^cd^	1.52^c–f^	1.48^c–g^	1.54^cde^
	F	1.24^d^	1.28^cd^	1.39^cd^	1.19^g^	1.32^d–g^	1.24^efg^
	SFN	1.52^cd^	1.44^cd^	1.54^cd^	1.71^c^	1.37^d–g^	1.61^cd^
	FN	1.37^cd^	1.41^cd^	1.37^cd^	1.23^fg^	1.28^efg^	1.33^d–g^
	SEM	0.12			0.15		

*Note:* Within each storage condition (+4°C and −18°C), pH, a_w_, and TBARS values with different superscript letters are significantly different (*p* < 0.05). Comparisons were conducted separately within each storage condition. Superscript ranges were a–e and a–f for pH, a–f and a–e for a_w_, and a–d and a–g for TBARS at +4°C and −18°C, respectively.

Abbreviations: Control, sucuk döner kebab (without starter cultures and sodium nitrite); F, fermented sucuk döner kebab (addition of starter cultures and 72 h fermentation duration); FN, fermented sucuk döner kebab with 100 ppm added sodium nitrite; SEM, standard error of the mean; SF, semi‐fermented sucuk döner kebab (addition of starter cultures and 24 h fermentation duration); SFN, semi‐fermented sucuk döner kebab with 100 ppm added sodium nitrite.

Water activity (a_w_) values (Table 2) ranged between 0.797 and 0.847 among treatments on processing day. While the lowest a_w_ was found in F treatment, the highest a_w_ values were found in SF and SFN treatments (*p* < 0.05). Fermentation is a process that typically leads to a reduction in a_w_ due to the conversion of carbohydrates into lactic acid, which lowers pH and can affect water binding in the muscle foods (Özer and Kılıç 2021). The low a_w_ observed in F treatment is likely a result of moisture loss during fermentation, as the activity of starter cultures can cause moisture migration and binding changes (Zhou et al. 2023). In contrast, semi‐fermented treatments (SF and SFN) showed higher a_w_ values, which may be related to less extensive fermentation and thus lower starter culture activity during the initial processing phase, resulting in less moisture removal from the sucuk döner kebabs. In general, a_w_ values stayed consistent throughout 30 days storage at +4°C, in contrast to the control and F treatment, which exhibited a progressive increase (*p* < 0.05). Upon completion of 30 days storage at both +4°C and −18°C, the lower a_w_ values were determined in F treatment compared to control, SF and SFN (*p* < 0.05). This could be attributed to the higher fermentation rates and the lower moisture retention characteristic in F treatment, which could cause more moisture to be lost during storage. With respect to frozen storage, the lowest a_w_ values were determined in F treatment (*p* < 0.05) as determined in refrigerated storage at the end of 30 days storage. FN treatment had the lower a_w_ compared to control, SF and SFN (*p* < 0.05).

The results (Table 3) on L*, a* and b* values reflected significant differences in color characteristics across sucuk döner kebab treatments during processing and storage. These differences were influenced by factors such as nitrite addition, fermentation, and storage conditions. When sucuk döner kebab treatments prepared for +4°C storage are compared, in general, there was no L* value difference among most of the treatments on processing day, but SF treatment had lower L* than F treatment (*p* < 0.05). L* values remained unchanged during storage (+4°C) among most of treatments except SF treatment where L* values increased during the same period of time (*p* < 0.05). An increase in lightness during storage could be attributed to protein denaturation and fat oxidation, which can occur as the product interacts with the storage environment (Guyon et al. 2016). In general, no differences were also observed among most of the treatments at the end of refrigerated storage. Only SF treatment had higher (*p* < 0.05) L* values than control. There was also no L* values difference on processing day among treatments prepared for frozen storage. During frozen storage, L* values showed an increase (*p* < 0.05) in SF, F and FN treatments. This results in accordance with previous study that indicated an icrease in L* and a decrease in a* and b* values of frozen minced beef products due to pigment oxidation enhanced by ice crystallization during frozen storage (Lee et al. 2021). At the end of frozen storage, F treatment had higher (*p* < 0.05) L* values than SFN treatment and control. As far as a* values are concerned, a* values of SFN and FN treatments prepared for both refrigerated and frozen storage were higher (*p* < 0.05) than others on processing day. The addition of nitrite generally enhances the redness of cured meat products by stabilizing the myoglobin in its nitrosomyoglobin form and this effect is especially visible in nitrite‐containing treatments (SFN and FN), where nitrite may help to maintain the red color of the meat throughout the fermentation and storage process, preventing oxidation of myoglobin (Higuero et al. 2020). During both refrigerated and frozen storage, a* values decreased in F, SFN and FN treatments (*p* < 0.05). This reduction in redness over time is likely due to oxidation of myoglobin during storage (Lee et al. 2021). However, SFN and FN treatments maintained higher (*p* < 0.05) a* values at the end of both refrigerated and frozen storage compared to other treatments, indicating that nitrite helped preserve the redness of the sucuk döner kebabs even after storage. b* values in SF and SFN treatments prepared for both storage temperatures were lower (*p* < 0.05) than others on processing day. Over refrigerated storage, b* values generally decreased (*p* < 0.05) in all treatments. Meantime, b* values of the most treatments generally did not change during frozen storage. SF treatment exhibited lower b* values compared to both control and F treatment after refrigerated storage for 30 days, whereas SFN displayed lower b* values than control, SF and F treatments upon the conclusion of the frozen storage period (*p* < 0.05).

**TABLE 3:** CIE color values of the cooked sucuk döner kebab treatments stored at 4°C (A) and −18°C (B).

	Treatments	L*			a*			b*		
		0 days	15 days	30 days	0 days	15 days	30 days	0 days	15 days	30 days
Refrigerated Storage (A)	Control	41.0^de^	45.0^a^	41.2^de^	11.8^efg^	10.4^g^	11.8^efg^	10.9^ab^	11.5^a^	8.3^d–g^
	SF	39.6^e^	44.0^abc^	43.7^abc^	11.2^efg^	11.1^fg^	12.2^ef^	8.3^def^	9.6^bcd^	6.5^h^
	F	42.2^cd^	43.0^a–d^	42.5^bcd^	12.9^e^	11.9^efg^	10.4^g^	9.8^bc^	9.5^bcd^	9.3^cde^
	SFN	41.0^de^	42.6^bcd^	42.2^cd^	24.2^b^	21.8^cd^	21.1^d^	7.7^fgh^	7.2^fgh^	6.8^gh^
	FN	41.0^de^	44.5^ab^	42.8^a–d^	27.5^a^	23.1^bc^	20.3^d^	9.8^bc^	7.0^fgh^	7.8^e–h^
	SEM	0.77			0.61			0.58		
Frozen Storage (B)	Control	42.4^def^	43.5^cde^	43.9^bcd^	12.0^def^	10.0^g^	12.6^de^	11.0^bc^	13.3^a^	11.1^b^
	SF	40.6^f^	45.5^abc^	46.3^ab^	10.7^fg^	15.1^c^	13.1^d^	8.4^ef^	9.5^b–e^	9.4^cde^
	F	43.3^c–f^	46.5^ab^	47.3^a^	13.6^cd^	10.8^efg^	10.8^efg^	10.3^bcd^	11.0^bc^	9.5^b–e^
	SFN	40.9^ef^	44.4^bcd^	43.3^c–f^	26.5^a^	21.6^b^	21.2^b^	8.3^ef^	7.3^f^	7.3^f^
	FN	42.4^def^	47.6^a^	45.9^abc^	25.9^a^	22.1^b^	21.8^b^	9.8^b–e^	9.4^de^	8.61^ef^
	SEM	1.00			0.67			0.61		

*Note:* Within each storage condition (+4°C and −18°C), L*, a*, and b* values with different superscript letters are significantly different (*p* < 0.05). Comparisons were conducted separately within each storage condition. Superscript ranges were a–e and a–f for L*, a–g and a–g for a*, and a–h and a–f for b* at +4°C and −18°C, respectively.

Abbreviations: Control, sucuk döner kebab (without starter cultures and sodium nitrite); F, fermented sucuk döner kebab (addition of starter cultures and 72 h fermentation duration); FN, fermented sucuk döner kebab with 100 ppm added sodium nitrite; SEM, standard error of the mean; SF, semi‐fermented sucuk döner kebab (addition of starter cultures and 24 h fermentation duration); SFN, semi‐fermented sucuk döner kebab with 100 ppm added sodium nitrite.

Texture profile analysis results (Table 4) indicated that fermentation, fermentation duration, and nitrite addition exerted limited but treatment‐dependent effects on the textural properties of cooked sucuk döner kebabs. Under refrigerated storage, FN samples exhibited higher hardness and chewiness values at day 0, whereas FN and SFN treatments showed relatively higher cohesiveness values than the other treatments (*p* < 0.05). Under frozen storage, F and FN treatments showed higher hardness values at day 0, while F samples exhibited the greatest hardness and chewiness values at day 30 compared to other treatments (*p* < 0.05). The increase in these parameters may be associated with protein structural modifications and moisture redistribution occurring during fermentation. In contrast, FN exhibited lower (*p* < 0.05) cohesiveness and resilience than the other treatments at day 30. These differences may be related to starter culture activity, which can influence protein modifications and pH reduction during fermentation. Marcos et al. (2007) similarly reported that starter culture application increased cohesiveness, chewiness, and springiness in fermented sausages, mainly due to proteolytic activity and pH changes, with textural parameters increasing as pH approached the isoelectric point of meat proteins.

**TABLE 4:** Texture profile analysis results of the cooked sucuk döner kebab treatments stored at +4°C (A) and −18°C (B).

(A) Treatments	Hardness (N)			Adhesiveness (mJ)			Resilience		
	0 days	15 days	30 days	0 days	15 days	30 days	0 days	15 days	30 days
Control	4.51^c^	5.46^abc^	6.10^a^	0.28^abc^	0.15^bc^	0.10^bc^	0.11^e^	0.19^ab^	0.17^abc^
SF	4.78^c^	4.83^c^	5.14^abc^	0.13^bc^	0.28^abc^	0.23^abc^	0.17^abc^	0.20^a^	0.13^cde^
F	5.26^abc^	5.30^abc^	4.81^c^	0.13^bc^	0.33^ab^	0.13^bc^	0.16^a–d^	0.12^de^	0.13^de^
SFN	4.39^c^	4.98^bc^	4.58^c^	0.05^c^	0.20^abc^	0.40^a^	0.19^ab^	0.19^ab^	0.12^e^
FN	6.04^ab^	5.24^abc^	5.34^abc^	0.15^bc^	0.05^c^	0.20^abc^	0.15^b–e^	0.18^ab^	0.14^cde^
SEM	0.39			0.08			0.02		

Treatments	Cohesiveness			Springiness			Chewiness (N)		
	0 days	15 days	30 days	0 days	15 days	30 days	0 days	15 days	30 days
Control	0.57^b^	0.62^ab^	0.57^b^	0.89^abc^	0.87^abc^	0.86^abc^	2.43^bcd^	2.94^abc^	3.00^abc^
SF	0.61^ab^	0.62^ab^	0.59^ab^	0.89^ab^	0.92^a^	0.87^abc^	2.67^a–d^	3.10^ab^	2.64^a–d^
F	0.63^ab^	0.60^ab^	0.60^ab^	0.90^ab^	0.81^bc^	0.83^abc^	2.91^abc^	2.56^bcd^	2.41^cd^
SFN	0.67^a^	0.63^ab^	0.60^ab^	0.82^abc^	0.87^abc^	0.79^c^	2.38^cd^	2.71^a–d^	2.17^d^
FN	0.63^ab^	0.66^a^	0.56^b^	0.86^abc^	0.86^abc^	0.87^abc^	3.24^a^	2.96^abc^	2.58^a–d^
SEM	0.03			0.04			0.24		

(B) Treatments	Hardness (N)			Adhesiveness (mJ)			Resilience		
	0 days	15 days	30 days	0 days	15 days	30 days	0 days	15 days	30 days
Control	4.53^def^	4.16^f^	4.82^c–f^	0.30^ab^	0.30^ab^	0.20^b^	0.12^fg^	0.10^g^	0.16^bcd^
SF	4.83^c–f^	4.37^f^	4.68^c–f^	0.18^b^	0.38^ab^	0.18^b^	0.18^ab^	0.17^abc^	0.16^b–e^
F	5.29^b–e^	5.84^ab^	6.72^a^	0.15^b^	0.25^b^	0.35^ab^	0.17^abc^	0.15^c–f^	0.13^d–g^
SFN	4.39^ef^	4.20^f^	4.35^f^	0.08^b^	0.28^ab^	0.15^b^	0.20^a^	0.13^efg^	0.17^abc^
FN	6.09^ab^	5.41^bcd^	5.60^bc^	0.15^b^	0.65^a^	0.40^ab^	0.16^b–e^	0.12^fg^	0.07^h^
SEM	0.32			0.14			0.01		

Treatments	Cohesiveness			Springiness			Chewiness (N)		
	0 days	15 days	30 days	0 days	15 days	30 days	0 days	15 days	30 days
Control	0.57^bc^	0.51^cd^	0.63^ab^	0.88^a^	0.86^ab^	0.85^ab^	2.40^cde^	1.91^e^	2.57^cd^
SF	0.60^ab^	0.64^ab^	0.63^ab^	0.88^a^	0.86^ab^	0.88^a^	2.71^bcd^	2.41^cde^	2.54^cde^
F	0.62^ab^	0.62^ab^	0.59^ab^	0.91^a^	0.79^b^	0.87^ab^	2.91^abc^	2.43^cde^	3.44^a^
SFN	0.66^a^	0.58^bc^	0.66^a^	0.84^ab^	0.84^ab^	0.88^a^	2.45^cde^	1.92^e^	2.52^cde^
FN	0.64^ab^	0.51^cd^	0.46^d^	0.89^a^	0.83^ab^	0.84^ab^	3.24^ab^	2.34^cde^	2.15^de^
SEM	0.03			0.03			0.22		

*Note:* A^a–e^: For each textural attribute specified in the table (4°C), values superscripted with different letters are significantly different (*P* < 0.05). B^a–h^: For each textural attribute specified in the table (−18°C), values superscripted with different letters are significantly different (*p* < 0.05).

Abbreviations: Control, sucuk döner kebab (without starter cultures and sodium nitrite); F, fermented sucuk döner kebab (addition of starter cultures and 72 h fermentation duration); FN, fermented sucuk döner kebab with 100 ppm added sodium nitrite; SEM, standard error of the mean; SF, semi‐fermented sucuk döner kebab (addition of starter cultures and 24 h fermentation duration); SFN, semi‐fermented sucuk döner kebab with 100 ppm added sodium nitrite.

Results (Table 1) indicated that moisture, fat, protein, and ash contents of sucuk döner kebab treatments ranged between 41.46%–47.24%, 19.56%–22.92%, 29.13%–32.54%, and 3.48%–4.15% among treatments, respectively. In general, even though there were no moisture differences among most of the treatments, the SFN treatment had higher moisture content than the SF and F treatments (*p* < 0.05). The lowest fat content was observed in the control (*p* < 0.05). Among fermented and semi‐fermented treatments, only the fat difference was evident between the FN and SFN treatments (*p* < 0.05). No significant protein content difference existed among treatments. Regarding ash content, the SFN treatment had lower ash content compared to the control, SF, and F treatments (*p* < 0.05).

### 3.3 TBARS

TBARS results (Table 2) indicated that application of fermentation, regardless of fermentation duration or nitrite addition, contributed to a reduction (*p* < 0.05) in TBARS formations compared to control on processing day. While the highest (*p* < 0.05) TBARS values were found in control, the rest of the sucuk döner kebab treatments had similar TBARS levels. Compared to control, TBARS reduction rates determined on processing day were 37.55%, 47.68%, 35.86%, and 42.19% in SF, F, SFN, and FN treatments respectively. Our findings indicated that fermentation can act as a protective measure against lipid oxidation. The lower TBARS values observed in fermented treatments may be associated with LAB activity during fermentation. Previous studies have reported that LAB may contribute to oxidative stability in fermented meat products through fermentation‐related metabolic activities and the formation of antioxidant compounds and bioactive peptides (Astandifiyah et al. 2024; Łepecka et al. 2024). Therefore, the reduced lipid oxidation observed in fermented treatments could be partially attributed to metabolic changes occurring during fermentation. The enzymes derived from LAB, such as superoxide dismutase, catalase, and glutathione peroxidase, have also been reported to also play a vital role in reducing the peroxide content to control the development of lipid oxidation (Stadnik et al. 2022). Furthermore, it was noted that the fermented treatments with nitrite (SFN and FN) did not show significantly lower TBARS values compared to treatments without nitrite (SF and F). Even though TBARS values increased (*p* < 0.05) in control during 30 days refrigerated storage, TBARS values were quite stable in the rest of the sucuk döner kebab treatments. This result reinforced the idea that fermentation provided a degree of protection against oxidative deterioration during storage. This is in line with previous studies indicating that lactic acid bacteria involved in fermentation can have antioxidant properties via producing antioxidant metabolites and exhibiting free radical scavenging and metal ion chelating abilities (Astandifiyah et al. 2024). At the end of refrigerated storage, all fermented sucuk döner kebab samples had similar TBARS to each other but lower (*p* < 0.05) TBARS values than control as described for processing day. As far as frozen storage is concerned, a similar trend mentioned for sucuk döner kebabs produced for refrigerated storage existed, but the only difference noticed was that F treatment had lower (*p* < 0.05) TBARS values than control, SF, and SFN treatments. TBARS values determined in all sucuk döner kebab samples did not change during the frozen storage period. At the end of frozen storage, the highest (*p* < 0.05) TBARS were in control and the rest of the sucuk döner kebab treatments had similar TBARS levels.

### 3.4 Residual Nitrite

**FIGURE 2:**
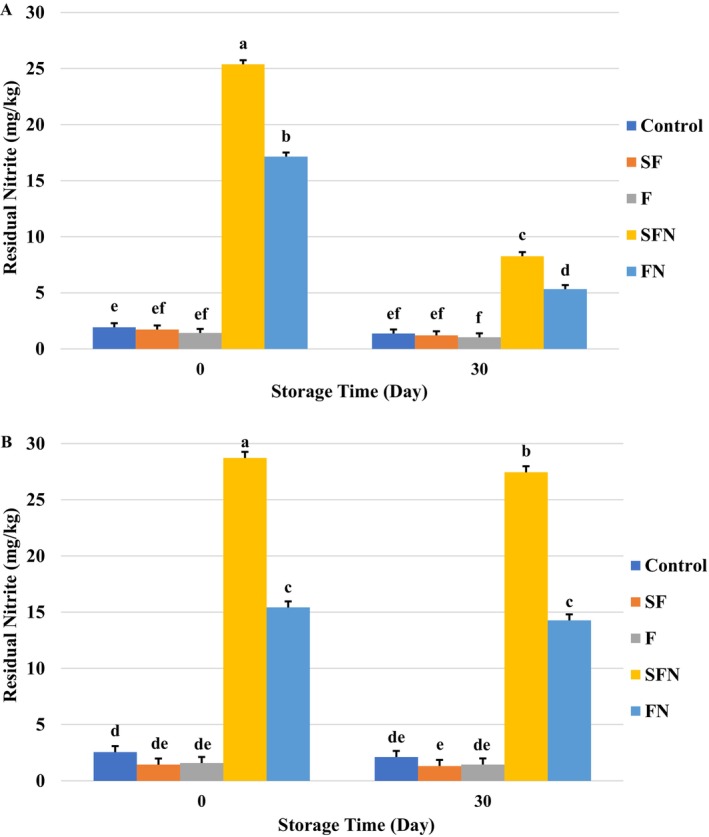
Residual nitrite levels (mg/kg) of the cooked sucuk döner kebab treatments stored at 4°C (A) and −18°C (B). F, Fermented sucuk döner kebab (addition of starter cultures and 72 h fermentation duration); FN, Fermented sucuk döner kebab with 100 ppm added sodium nitrite; SEM, Standard error of the mean; SF, Semi‐fermented sucuk döner kebab (addition of starter cultures and 24 h fermentation duration); SFN, Semi‐fermented sucuk döner kebab with 100 ppm added sodium nitrite. (A ^a–f^) Within a figure, values superscripted with different letters are significantly different (*p* < 0.05); B^a–e^Within a figure, values superscripted with different letters are significantly different (*p* < 0.05).

Residual nitrite results (Figure 2) provided valuable insights into the residual nitrite in sucuk döner kebabs, particularly in relation to the fermentation process. Results revealed no significant difference in residual nitrite levels in control and the sucuk döner treatments without added nitrite (SF and F), indicating that the residual nitrite levels were unaffected by the fermentation process when no nitrite was added. The amount of residual nitrite determined in these treatments was also the lowest residual nitrite levels among all treatments (*p* < 0.05). Residual nitrite levels in these treatments did not undergo significant changes over 30 days storage period at both storage temperatures, and the residual nitrite levels were also similar among these three sucuk döner kebab treatments at the end of 30 days storage at both storage temperatures.

The research results showed that fermentation significantly impacted the residual nitrite levels in sucuk döner kebab manufactured with nitrite (*p* < 0.05). In general, SFN treatment had the highest (*p* < 0.05) residual nitrite levels and this treatment was followed by FN treatment. This difference between these two treatments is thought to be associated with less extensive fermentation that took place in semi‐fermented nitrite‐added sucuk döner samples. Some previous studies have suggested that certain lactic acid bacteria activities during fermentation, particularly those with nitrite reductase and heme‐independent nitrite reductase, reduce the residual nitrite levels in fermented muscle foods (Zhu et al. 2020). Lactic acid bacteria and other microorganisms involved in fermentation might contribute to the reduction of added nitrite to nitrous acid or nitric oxide during fermentation, although these processes tend to be more pronounced in longer fermentation periods (Wang et al. 2013). During fermentation, pH reduction driven by the activity of lactic acid bacteria also plays a crucial role in facilitating the conversion of nitrite into nitrous acid and nitric oxide, leading to a notable reduction in the residual nitrite levels (Sebranek and Bacus 2007). The higher levels of residual nitrite in semi‐fermented treatments compared to fermented treatments in the present study may be due to the difference in fermentation time duration, because the fermentation was not extensive enough in the semi‐fermented treatments to significantly reduce nitrite. On the other hand, semi‐fermented and fermented treatments with added nitrite (SFN and FN) showed differing residual nitrite levels, particularly in relation to storage temperature. Results indicated that the storage temperature also played a crucial role in the levels of residual nitrite, where frozen storage effectively inhibited nitrite reduction. In contrast, the lower temperatures associated with refrigeration likely slowed down the rate of nitrite depletion but did not completely stop it. No significant changes were observed in the residual nitrite levels in most of the treatments during the 30 days storage period at −18°C, but the residual nitrite level in SFN treatment decreased during the same period of time. It has been suggested that freezing significantly slows down the activity of all microorganisms including nitrite‐reducing bacteria (Dave and Ghaly 2011). As freezing effectively prevents microbial activity, it would prevent the reduction of nitrite, thus maintaining higher levels of residual nitrite in the semi‐fermented (SFN) samples during storage. At the end of both frozen and refrigerated storage, the residual nitrite level detected in FN treatment was still lower than the residual nitrite level detected in SFN treatment (*p* < 0.05).

### 3.5 Microbiological Results

Results (Table 5) indicated that the highest TMAB count was obtained in SFN treatment on processing day for both samples prepared for refrigerated and frozen storage (*p* < 0.05). In general, regardless of nitrite addition, TMAB count increased (*p* < 0.05) in all semi‐fermented and fermented sucuk döner kebab treatments during both refrigerated and frozen storage, whereas TMAB was quite stable in control at duration of both refrigerated and frozen storage. At the end of refrigerated storage, the highest (*p* < 0.05) TMAB counts were detected in SF and SFN treatments and control had the lowest TMAB (*p* < 0.05). On the other hand, while the highest (*p < 0.05*) TMAB was obtained in F and FN treatments at the end of frozen storage, other treatments had similar TMAB count.

**TABLE 5:** Microbiological analysis results (Log CFU/g) of the cooked sucuk döner kebab treatments stored at 4°C and −18°C.

	Groups	Storage time (days; +4°C)			Storage time (days; −18°C)		
		0 days	15 days	30 days	0 days	15 days	30 days
TMAB	Control	4.26^g^	4.12^gh^	4.24^g^	4.26^fg^	4.34^ef^	4.30^ef^
	SF	3.93^h^	6.30^b^	7.49^a^	3.93^h^	4.70^cd^	4.54^de^
	F	4.00^gh^	5.38^e^	6.28^b^	4.01^gh^	4.90^bc^	5.14^ab^
	SFN	4.73^f^	5.92^cd^	7.55^a^	4.71^cd^	4.35^ef^	4.35^ef^
	FN	4.01^gh^	5.64^de^	6.05^bc^	4.01^gh^	5.24^a^	5.04^ab^
	SEM	0.11			0.10		
Total coliforms	Control	3.74^abc^	3.60^a–d^	3.56^bcd^	3.74^abc^	3.61^abc^	3.62^abc^
	SF	3.55^bcd^	4.00^a^	2.88^e^	3.60^abc^	3.59^abc^	3.48^abc^
	F	3.39^cd^	3.20^de^	3.36^cd^	3.43^a–d^	3.54^abc^	3.65^abc^
	SFN	3.77^abc^	3.21^de^	3.27^de^	3.80^ab^	3.32^cd^	3.00^d^
	FN	3.85^ab^	3.50^bcd^	3.76^abc^	3.84^a^	3.30^cd^	3.39^bcd^
	SEM	0.15			0.16		
LAB	Control	< 1.00^j^	4.28^ghi^	4.70^fg^	< 1.00^h^	< 1.00^h^	< 1.00^h^
	SF	3.86^i^	6.54^b^	8.34^a^	3.81^fg^	4.38^cd^	4.54^bcd^
	F	4.25^hi^	4.95^ef^	5.67^cd^	4.23^de^	4.51^cd^	4.60^bc^
	SFN	4.52^fgh^	5.79^c^	8.70^a^	4.60^bc^	3.68^g^	4.06^ef^
	FN	4.87^ef^	5.25^de^	5.79^c^	4.84^ab^	4.99^a^	4.60^bc^
	SEM	0.22			0.13		
*Lactococcus‐Streptococcus* spp.	Control	4.21^ef^	4.72^cde^	4.07^f^	4.20^a–e^	4.39^ab^	4.17^a–e^
	SF	4.35^ef^	5.21^abc^	5.36^ab^	4.28^a–d^	4.07^de^	4.19^a–e^
	F	4.00^f^	5.42^ab^	5.67^a^	3.98^e^	4.34^abc^	4.13^b–e^
	SFN	4.38^def^	4.95^bc^	4.91^bcd^	4.42^a^	4.21^a–e^	4.20^a–e^
	FN	4.12^f^	5.46^ab^	5.73^a^	4.11^cde^	4.22^a–e^	4.00^e^
	SEM	0.22			0.09		

*Note:* Within each storage condition (+4°C and −18°C), TMAB, total coliforms, LAB, and *Lactococcus‐Streptococcus* spp. counts with different superscript letters are significantly different (*p* < 0.05). Comparisons were conducted separately within each storage condition. Superscript ranges were a–h and a–h for TMAB, a–e and a–d for total coliforms, a–h and a–h for LAB, and a–f and a–e for *Lactococcus‐Streptococcus* spp. at +4°C and −18°C, respectively.

Abbreviations: Control, sucuk döner kebab (without starter cultures and sodium nitrite); F, fermented sucuk döner kebab (addition of starter cultures and 72 h fermentation duration); FN, fermented sucuk döner kebab with 100 ppm added sodium nitrite; LAB, Lactic acid bacteria; SEM, standard error of the mean; SF, semi‐fermented sucuk döner kebab (addition of starter cultures and 24 h fermentation duration); SFN, semi‐fermented sucuk döner kebab with 100 ppm added sodium nitrite; TMAB, Total mesophilic aerobic bacteria.

As far as total coliform bacteria are concerned, all sucuk döner kebab samples prepared for both storage types had similar total coliform bacteria counts on processing day. In general, total coliform bacteria counts did not change in sucuk döner samples during both refrigerated and frozen storage periods. However, a significant decrease (*p* < 0.05) in total coliform bacteria was observed in SF and SFN treatments during refrigerated storage, while a similar reduction was obtained in SFN and FN treatments during frozen storage. The decrease in coliform counts in frozen storage could reflect the inhibitory effects of freezing temperatures on coliforms, as cold storage generally slows down bacterial growth significantly (Medić et al. 2018; Mohammed et al. 2021). At the end of refrigerated storage, the lowest total coliform bacteria count was detected in the SF treatment. On the other hand, the SFN treatment had a lower total coliform bacteria count compared to the control, SF, and F treatments at the end of the frozen storage period (*p* < 0.05).

Regarding LAB count, as expected, control sucuk döner kebab samples prepared for both storage temperatures had < 1.00 log CFU/g LAB count on processing day. Even though LAB count in control was quite stable during frozen storage, it increased (*p* < 0.05) during refrigerated storage. On processing day, FN treatment prepared for refrigerated storage had higher LAB counts compared to SF and F treatments, whereas LAB counts of SFN treatment were similar to F and FN treatments but higher (*p* < 0.05) than SF treatment. At the same time, SFN and FN treatments prepared for frozen storage had the highest LAB counts, whereas these treatments were followed by F and SF treatments (*p* < 0.05). All treatments showed an increasing trend (*p* < 0.05) in LAB counts during refrigerated storage. This increasing trend was only evident in SF and F treatments in frozen storage where LAB counts were decreased (*p* < 0.05) in SFN treatment and were stable in FN treatment during the same period of time. The increase in LAB counts in all treatments during refrigerated storage is consistent with findings from previous studies that noted an increase in LAB populations during the ripening or fermentation of sausages (Casaburi et al. 2008). At the end of refrigerated storage, the highest (*p* < 0.05) LAB counts were obtained in SF and SFN treatments and these treatments were followed by FN, F and control. At the end of frozen storage, SF, F and FN treatments had higher LAB counts than SFN treatment which had higher LAB counts compared to control (*p* < 0.05). Ibrahim (2013) demonstrated that lactic acid produced by LAB lowers pH of the food matrix, thereby creating self‐limiting conditions that can inhibit further LAB growth. Authors also stated that most LAB genera exhibit optimal metabolic function at a pH of 5–6, with significant growth suppression occurring at or below pH 4.4. In our study, it is thought that the extent of fermentation in semi‐fermented sucuk döner kebabs was limited, resulting in moderate acidification, thus pH drop was insufficient to reach the inhibitory levels typically observed in fermented samples.

*Lactococcus‐Streptococcus* spp. results revealed that all treatments prepared for refrigerated storage had similar *Lactococcus‐Streptococcus* spp. counts on processing day. Regarding sucuk döner kebabs prepared for frozen storage, even though *Lactococcus‐Streptococcus* spp. counts were generally similar among treatments on processing day, the SFN treatment had higher (*p* < 0.05) counts than the F and FN treatments. Although *Lactococcus‐Streptococcus* spp. counts obtained in all treatments did not show any changes during frozen storage, an increase (*p* < 0.05) in *Lactococcus‐Streptococcus* spp. was observed in the SF, F, and FN treatments during refrigerated storage. After 30 days of refrigerated storage, the F and FN treatments had higher (*p* < 0.05) *Lactococcus‐Streptococcus* spp. counts compared to the SFN treatment and control. The lowest *Lactococcus‐Streptococcus* spp. counts were obtained in the control at the end of refrigerated storage (*p* < 0.05). Upon completion of frozen storage, there were no differences among all sucuk döner kebabs regarding *Lactococcus‐Streptococcus* spp. counts. The relatively high *Lactococcus–Streptococcus* counts observed in the F and FN treatments after refrigerated storage suggest that fermentation did not effectively suppress the survival of these microorganisms under the applied conditions. This may be related to the acid tolerance and adaptation of these microorganisms to fermented meat environments, enabling their persistence during ripening and storage. Previous studies have similarly reported the continued presence of coccoid LAB throughout fermented sausage processing and storage (Drosinos et al. 2005; Casaburi et al. 2007). Furthermore, refrigerated storage may reduce microbial metabolic activity while not completely inhibiting the survival of these microorganisms.

### 3.6 Volatile Profile

Results (Table 6) revealed a total of 44 volatile compounds (8 aldehydes, 1 acid, 1 ketone, 17 terpenes, 6 sulfur compounds, 1 ester, 2 alcohols, 2 hydrocarbons, and 6 miscellaneous compounds) across the different treatments; however, the dominant volatile compounds in all sucuk döner kebab treatments were found to be 2‐methyl‐1‐butanol, allyl mercaptan, γ‐terpinene, allyl disulfide, cymol, acetic acid, methyl allyl disulfide, acetaldehyde, ethanol, limonene, cuminaldehyde, and caryophyllene.

**TABLE 6:** Volatile compounds of the cooked sucuk döner kebab treatments (peak area × 10^−6^).

Volatile compounds	Treatments					
	Control	SF	*F*	SFN	FN	SEM
**Acids**						
Acetic acid	ND	ND	6.83^a^	ND	6.95^a^	0.90
Subtotal (%)	ND	ND	6.83	ND	6.95	
**Alcohols**						
Ethanol	1.19^d^	2.78^c^	4.30^a^	1.04^d^	3.47^b^	0.34
2‐Methyl‐1‐butanol	42.42^b^	ND	ND	45.96^a^	ND	5.80
Subtotal (%)	43.61	2.78	4.3	47	3.47	
**Aldehydes**						
Acetaldehyde	1.04^c^	1.60^b^	2.09^a^	0.59^d^	1.90^a^	0.15
3‐Methyl‐butanal	ND	ND	0.19^b^	ND	0.34^a^	0.04
Pentanal	ND	ND	ND	ND	1.66	0.18
Hexanal	0.28^c^	0.38^c^	4.27^a^	ND	0.62^b^	0.43
Heptanal	ND	ND	3.60	ND	ND	0.04
Nonanal	ND	0.92^a^	0.99^a^	ND	ND	0.13
Cuminaldehyde	12.80^a^	6.43^c^	5.02^d^	7.44^bc^	7.73^b^	0.71
2‐Caren‐10‐al	1.33^a^	0.86^c^	1.16^b^	0.94^c^	1.08^b^	0.05
Subtotal (%)	15.45	10.19	17.32	8.97	13.33	
**Esters**						
Ethyl hexanoate	ND	ND	0.26^a^	ND	ND	0.03
Subtotal (%)	ND	ND	0.26	ND	ND	
**Hydrocarbons**						
2‐Bromopropane	0.28	ND	ND	ND	ND	0.03
1‐Bromopropane	ND	ND	ND	0.32	ND	0.03
Subtotal (%)	0.28	ND	ND	0.32	ND	
**Ketones**						
2‐Propanone	2.49^b^	3.39^a^	0.65^d^	1.36^c^	1.41^c^	0.26
Subtotal (%)	2.49	3.39	0.65	1.36	1.41	
**Sulfur compounds**						
Methanethiol	ND	ND	ND	0.63	ND	0.07
Allyl mercaptan	ND	30.25^a^	16.82^b^	ND	14.72^b^	3.05
Allyl methyl sulfide	0.87^c^	1.20^b^	1.90^a^	0.37^e^	0.68^d^	0.14
Diallyl sulfide	0.67^c^	1.22^b^	1.52^a^	0.51^d^	1.56^a^	0.12
Methyl allyl disulfide	0.64^e^	1.73^c^	2.08^b^	0.85^d^	2.42^a^	0.19
Allyl disulfide	6.90^c^	11.06^b^	14.47^a^	7.93^c^	12.68^ab^	0.77
Subtotal (%)	9.08	45.46	36.79	10.29	32.06	
**Terpenes**						
α‐Thujene	0.10^b^	0.11^b^	0.30^a^	0.11^b^	0.13^b^	0.02
α‐Pinene	0.20^a^	0.27^a^	0.26^a^	0.25^a^	0.27^a^	0.01
Sabinene	0.19^c^	0.21^c^	0.52^a^	0.18^c^	0.37^b^	0.04
β‐Pinene	1.07^d^	1.50^b^	1.37^c^	1.17^d^	1.62^a^	0.06
Myrcene	1.91^bc^	2.40^a^	2.10^b^	1.68^d^	1.90^c^	0.07
L‐Phellandrene	0.80^d^	1.76^a^	1.54^b^	0.69^d^	1.21^c^	0.11
δ‐3‐Carene	0.30^d^	1.90^a^	0.49^c^	0.43^c^	0.62^b^	0.16
α‐Terpinene	0.12^c^	0.23^b^	0.29^ab^	0.24^b^	0.32^a^	0.02
Cymol	7.07^d^	9.95^b^	8.99^c^	9.96^b^	12.68^a^	0.49
Limonene	2.55^b^	3.75^a^	3.65^a^	2.74^b^	3.25^ab^	0.15
γ‐Terpinene	9.40^c^	12.28^ab^	11.36^bc^	9.85^c^	14.38^a^	0.51
α‐Terpinolene	ND	ND	0.14	ND	ND	0.02
Linalool	0.36^c^	0.40^bc^	0.53^b^	0.49^bc^	0.81^a^	0.04
4‐terpineol	ND	ND	0.32^a^	0.21^b^	0.28^a^	0.04
α‐Copaene	0.48^bc^	0.41^c^	0.56^b^	0.43^c^	0.78^a^	0.04
Caryophyllene	2.77^b^	2.43^c^	3.28^a^	2.23^c^	3.54^a^	0.13
Cedr‐8‐ene	0.48^b^	0.36^c^	0.57^a^	0.41^bc^	0.63^a^	0.03
Subtotal (%)	27.08	37.96	36.27	31.07	42.79	
**Miscellaneous compounds**						
Carbon disulfide	ND	ND	ND	0.15	ND	0.02
2,5‐Octanedione	ND	ND	1.60	ND	ND	0.02
1.8‐Cineole	0.12^b^	ND	ND	0.21^a^	ND	0.02
Phellandral	0.56^a^	0.22^b^	ND	0.33^b^	ND	0.06
Methyl eugenol	0.41^a^	ND	0.17^c^	0.30^b^	ND	0.04
cis‐Caryophyllene	0.17	ND	ND	ND	ND	0.02
Subtotal (%)	1.26	0.22	1.77	0.99	0	

*Note:* ^a–c^For each volatile compound specified within a table, values superscripted with different letters are significantly different (*p* < 0.05).

Abbreviations: F, fermented sucuk döner kebab (addition of starter cultures and 72 h fermentation duration); FN, fermented sucuk döner kebab with 100 ppm added sodium nitrite; ND, not detected; SEM, standard error of the mean; SF, semi‐fermented sucuk döner kebab (addition of starter cultures and 24 h fermentation duration); SFN, semi‐fermented sucuk döner kebab with 100 ppm added sodium nitrite.

Our findings indicated that terpenes were the dominant compounds identified in sucuk döner kebab treatments. Myrcene content was the highest in SF compared to the rest of the treatments (*p* < 0.05). The highest cymol content was observed in the FN treatment followed by SFN, SF, and F treatments, whereas the lowest level was found in the control (*p* < 0.05). Limonene contents found in SF and F treatments were higher than those found in the control and SFN (*p* < 0.05). γ‐terpinene content of the FN treatment was higher than that of the control, F, and SFN treatments and similar to that found in the SF treatment (*p* < 0.05). The highest caryophyllene contents were determined in the F and FN treatments followed by the control, SF, and SFN treatments (*p* < 0.05). Terpenes have generally been reported as the most abundant volatile compounds in fermented muscle foods and they are originated from added spices and sulfur‐ and nitrogen‐containing compounds derived from Maillard reactions (Montanari et al. 2016). Previous studies also reported terpenes as the main aroma compounds followed by acids, ketones, alcohols, esters, hydrocarbons, and miscellaneous compounds in Macedonian and Turkish fermented sausages (Sulejmani and Demiri 2019). These compounds are typically produced during the fermentation process or as a result of reactions such as lipid oxidation and contribute to the characteristic flavors of fermented muscle foods (Kosowska et al. 2017). The highest cumin aldehyde level existed in the control followed by FN, SFN, SF, and F treatments, respectively (*p* < 0.05). Cumin aldehyde, a key aromatic compound derived from cumin, has been previously identified in fermented sucuk, contributing to its distinctive flavor profile. Cumin aldehyde is derived from cumin and previously determined in fermented sucuk (Ercoşkun 1999). The highest hexanal content was found in the F treatment followed by FN, SF, and control (*p < 0.05*). Hexanal was not determined in the SFN treatment. Hexanal is known as an indicator of lipid oxidation; therefore, elevated levels are associated with flavor deterioration and the development of a rancid aroma in meat products (Ramírez and Cava 2007). Results indicated that nitrite addition resulted in a reduction in hexanal level in both semi‐fermented and fermented sucuk döner kebabs (*p* < 0.05). Heptanal, another aldehyde compound, was exclusively detected in the F treatment. The highest acetaldehyde content was found in fermented sucuk döner kebab treatments with or without nitrite (F and FN) followed by SF, control, and SFN (*p* < 0.05). These treatments (F and FN) also had higher ethanol content compared to others, and the highest level was found in the F treatment (*p* < 0.05). It has been reported that ethanol was the most abundant alcohol in the fermented sausages and could be produced by lactose metabolism or acetaldehyde reduction (Sulejmani and Demiri 2019). Ethanol was also stated as the main reason for the elevation in alcohol contents of beef across the storage and a contributor to the grilled aroma characteristic (Ismail et al. 2008; Mottram 1998). Even though 2‐methyl‐1‐butanol was not found in SF, F, and FN treatments, it was the highest volatile compound existing in SFN and the control (*p* < 0.05). Furthermore, the 2‐methyl‐1‐butanol level was even higher in the SFN treatment compared to the control (*p* < 0.05). It has been stated that alcohols like ethanol and 2‐methyl‐1‐butanol are primarily produced through the chemical reduction of aldehydes, lipid oxidation, and the catabolism of amino acids (Ao et al. 2022).

2‐propanone content was higher in SF treatment and lower in F, SFN, and FN treatments compared to control (*p* < 0.05). Ketones were reported to be probably generated via both biochemical pathways and thermal processing (Lorenzo et al. 2014). Acetic acid was only found in F and FN treatments. Acetic acid, a contributor to the aroma of fermented sausages, is primarily produced during fermentation through lipolysis of triglycerides. It may also result from citrate or lactate fermentation, amino acid catabolism, and lipid oxidation (Kaban 2010). In the present study, acetic acid was the only organic acid detected in the volatile profile. However, this does not indicate the absence of other organic acids, as compounds with limited volatility, such as lactic acid, may not be adequately represented in the volatile fraction (Olivares et al. 2015). Therefore, the relationship between LAB activity, organic acid production, and pH changes should be interpreted accordingly. Although allyl mercaptan was not found in control and SFN treatment, it was the predominant volatile compound in SF, F and FN treatments. The highest level was determined in SF treatment followed by F and FN treatment (*p* < 0.05). Methyl allyl disulfide content was higher in all fermented and semi‐fermented sucuk döner kebab treatments (SF, F, SFN and FN) compared to control and the highest content was found in FN treatment followed by F, SF and SFN treatments (*p* < 0.05). Allyl disulfide contents of SF, F and FN treatments were higher than those of control and SFN treatment (*p* < 0.05). Allyl disulfide was reported to be the most abundant compound responsible for distinct aroma flavors of cooked beef (Machiels et al. 2003). Sulfur compounds like allyl disulfide and methyl disulfide have been stated to be one of the main compounds in garlic (Rahman 2007).

### 3.7 Sensory Acceptance

**FIGURE 3:**
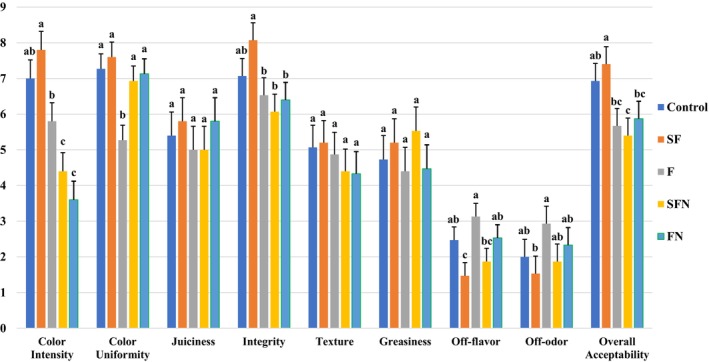
Sensory analysis results of the cooked sucuk döner kebab treatments. F, Fermented sucuk döner kebab (addition of starter cultures and 72 h fermentation duration); FN, Fermented sucuk döner kebab with 100 ppm added sodium nitrite; SF, Semi‐fermented sucuk döner kebab (addition of starter cultures and 24 h fermentation duration); SFN, Semi‐fermented sucuk döner kebab with 100 ppm added sodium nitrite. (^a–c^) For each sensory attribute specified within a figure, values superscripted with different letters are significantly different (*p* < 0.05).

Sensory evaluation results (Figure 3) showed that control and sucuk döner kebab treatments without nitrite (SF and F) had higher color intensity scores compared to the treatments with nitrite (SFN and FN, *p* < 0.05). This is consistent with the role of nitrite in meat products, which is known to affect the development of color due to its reaction with myoglobin, often leading to cured red color in the final product (Kilic et al. 2001). The color uniformity scores were similar across treatments except for F treatment, which received lower (*p* < 0.05) color uniformity score than other treatments. Panelist indicated no differences among the treatment regarding juiciness, integrity, texture and greasiness characteristics. In terms off‐flavor, F treatment was rated with a higher (*p* < 0.05) off‐flavor scores than SF and SFN treatments. Moreover, F treatment was also received a higher off‐odor scores than SF treatment (*p* < 0.05). As for as overall acceptability, the panelists assigned higher acceptability scores for semi‐fermented sucuk döner kebab without nitrite (SF), rating it as more acceptable than the other treatments (*p* < 0.05).

### 3.8 Principal Component Analysis Results

**FIGURE 4:**
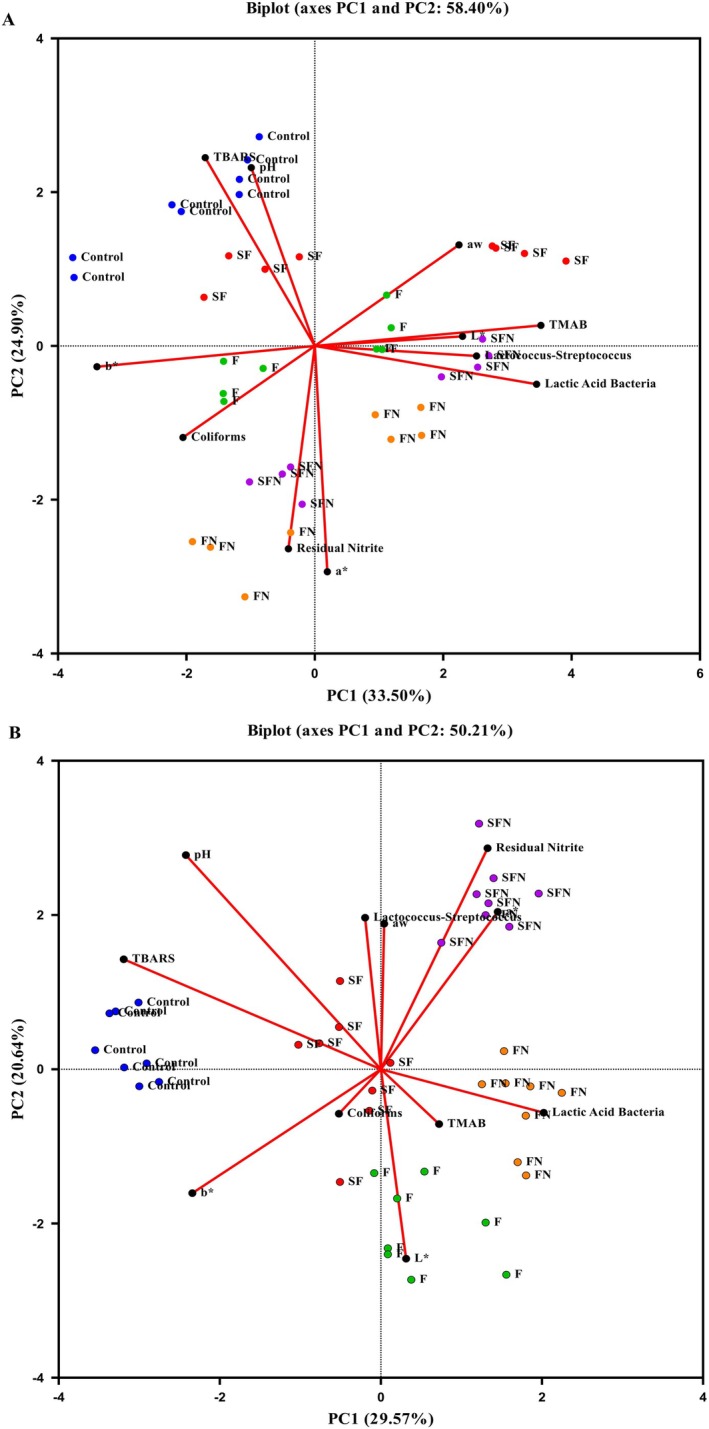
Biplot of principal component analysis for the physico‐chemical and microbiological parameters of the cooked sucuk döner kebabs stored at 4°C (A) and −18°C (B). a_w_, Water activity; F, Fermented sucuk döner kebab (addition of starter cultures and 72 h fermentation duration); FN, Fermented sucuk döner kebab with 100 ppm added sodium nitrite; SF, Semi‐fermented sucuk döner kebab (addition of starter cultures and 24 h fermentation duration); SFN, Semi‐fermented sucuk döner kebab with 100 ppm added sodium nitrite; TBARS, Hiobarbituric acid reactive substances; TMAB, Otal mesophilic aerobic bacteria.

The factor loadings of the first four principal components (Table 7) were taken to represent the interactions based on the Kaiser criterion stating eigenvalues greater than 1, while the biplot was generated using PC1 and PC2, which accounted for the greatest proportion of total variance and provided a clear visualization of treatment‐related separation. For the treatments stored at 4°C, the first two principal components (PCs) explained 58.40% of the total variability, with PC1 accounting for 33.50% and PC2 for 24.90%. The cumulative variance increased to 72.50% and 81.80% with the inclusion of PC3 and PC4, respectively. PC1 was mainly characterized by positive loadings of TMAB and lactic acid bacteria, while b* showed a negative association with this component. PC2 was mainly defined by a* and residual nitrite in the negative direction and by TBARS and pH in the positive direction. For the treatments stored at −18°C, PC1 and PC2 explained 50.21% of the total variability, with PC1 accounting for 29.57% and PC2 for 20.64%. The cumulative variance reached 65.66% with PC3 and 77.39% with PC4. PC1 was primarily associated with lactic acid bacteria and a* in the positive direction and TBARS and b* in the negative direction, whereas PC2 was mainly characterized by positive contributions of residual nitrite, pH, a_w_, and *Lactococcus–Streptococcus* spp., and a negative contribution of L*. The PC1–PC2 biplots (Figure 4) allowed visual discrimination of the treatments under both storage conditions. At 4°C, control was located close to TBARS and pH, indicating their association with oxidative and pH‐related changes, while SF was more related to a_w_ and L*. SFN was positioned near microbial variables, particularly TMAB, lactic acid bacteria, and *Lactococcus–Streptococcus* spp., whereas FN was separated toward a* and residual nitrite, suggesting a stronger relationship with a* and residual nitrite. At −18°C, control was associated with TBARS and pH related variation, whereas SF was mostly distributed near the center of the plot, indicating a less distinct relationship with a single parameter. SFN was grouped near residual nitrite, a*, a_w_, and *Lactococcus–Streptococcus* spp., while FN was mainly related to lactic acid bacteria, and F was closer to L*.

**TABLE 7:** Factor loadings of the significant principal components (PCs) for the physico‐chemical and microbiological parameters of the cooked sucuk döner kebabs stored at 4°C and −18°C.

	4°C				−18°C			
	PC1	PC2	PC3	PC4	PC1	PC2	PC3	PC4
TBARS	−0.368	0.623	−0.394	0.252	−0.833	0.339	−0.036	0.098
pH	−0.214	0.590	−0.676	−0.035	−0.632	0.660	−0.171	−0.229
a_w_	0.578	0.334	−0.579	0.048	0.018	0.518	−0.461	−0.489
L*	0.592	0.032	0.320	−0.179	0.141	−0.598	−0.501	−0.344
a*	0.050	−0.843	−0.437	−0.042	0.652	0.485	0.114	0.387
b*	−0.732	−0.078	0.426	0.114	−0.611	−0.391	−0.041	0.358
TMAB	0.906	0.068	0.005	0.195	0.325	−0.173	−0.753	0.052
Coliforms	−0.443	−0.342	−0.183	0.747	−0.136	−0.140	−0.500	0.664
*Lactococcus‐Streptococcus* spp.	0.648	−0.037	0.243	0.561	−0.051	0.537	−0.527	0.232
Lactic acid bacteria	0.889	−0.143	−0.117	−0.040	0.909	−0.137	−0.051	−0.050
Residual nitrite	−0.089	−0.758	−0.556	−0.116	0.595	0.681	0.055	0.156
Eigenvalue	4.349	3.236	1.839	1.215	3.219	2.248	1.700	1.290
Variance (%)	33.50	24.90	14.10	9.30	29.57	20.64	15.45	11.73
Cumulative (%)	33.50	58.40	72.50	81.80	29.57	50.21	65.66	77.39

Abbreviations: a_w_, Water activity; TBARS, Thiobarbituric acid reactive substances; TMAB, Total mesophilic aerobic bacteria.

## 4 Conclusion

Present study highlighted the critical influence of fermentation, fermentation duration, and nitrite addition on volatile profile, physico‐chemical, sensory, and microbiological properties of ready‐to‐eat (RTE) sucuk döner kebab. The findings demonstrated that fermentation significantly affected key parameters, such as pH, water activity, color, volatile profile, and lipid oxidation, with fermented products generally exhibiting lower pH, reduced a_w_, and greater protection against lipid oxidation. Sensory evaluations revealed that semi‐fermented sucuk döner kebab treatments without nitrite were most preferred by panelists for overall acceptability. In sucuk döner kebab processing, fermentation, regardless of its duration or whether curing was applied, consistently increased the counts of TMAB, LAB, and *Lactococcus‐Streptococcus* spp. at the end of 30 days of refrigerated storage. These results underscored the complex interactions between fermentation, nitrite addition, and storage conditions, providing valuable insights into optimizing sucuk döner kebab production for improved quality and preservation. It can be concluded that fermentation may be utilized in the processing of RTE sucuk döner kebab to improve shelf‐life, quality, and safety, while semi‐fermented sucuk döner kebab produced without nitrite may represent a promising processing approach for industrial application due to its high sensory acceptability and favorable quality characteristics.

## Author Contributions

**Birol Kılıç:** writing – original draft, writing – review and editing, project administration, methodology, investigation, conceptualization. **Azim Şimşek:** writing – review and editing, methodology, investigation, conceptualization. **Melis Yıldız:** writing – review and editing, methodology, investigation, conceptualization. **Ayşe Sinan:** funding acquisition, methodology, investigation, formal analysis, data curation.

## Funding

This study was supported by Türkiye Bilimsel ve Teknolojik Araştırma Kurumu (TUBITAK)‐Research Project Support Programme for Undergraduate Students under the Project number of 1919B012317916.

## Conflicts of Interest

The authors declare no conflicts of interest.

## Data Availability

The data that support the findings of this study are available from the corresponding author upon reasonable request.
